# Diagnostic journey and impact of enzyme replacement therapy for mucopolysaccharidosis IVA: a sibling control study

**DOI:** 10.1186/s13023-020-01618-y

**Published:** 2020-11-30

**Authors:** Can Ficicioglu, Dena R. Matalon, Nicole Luongo, Caitlin Menello, Tracy Kornafel, Andrew J. Degnan

**Affiliations:** 1 Division of Human Genetics/Metabolism, Lysosomal Storage Diseases Program, The Children’s Hospital of Philadelphia, Perelman School of Medicine, The University of Pennsylvania, 3401 Civic Center Blvd., Philadelphia, PA 19104 USA; 2 Stanford University, Lucile Packard Children’s Hospital, Palo Alto, CA USA; 3 Abington Hospital – Jefferson Health, Abington, PA USA

**Keywords:** Mucopolysaccharidosis IVA, Enzyme replacement therapy, Anterior beaking, Platyspondyly, Diagnosis, Treatment

## Abstract

**Background:**

Mucopolysaccharidosis (MPS) IVA, also known as Morquio A syndrome, is a rare autosomal recessive lysosomal storage disorder caused by a deficiency in the enzyme N-acetylgalactosamine-6-sulfatase. Early recognition, diagnosis, and treatment of this progressive, multisystem disease by enzyme replacement therapy (ERT) can lead to improved outcomes and reduced mortality.

**Methods:**

This report documents the diagnostic journey and treatment with ERT of three siblings with MPS IVA. Clinical outcome measures included growth, endurance, imaging, cardiac, respiratory, ophthalmology, and laboratory evaluations.

**Results:**

Three siblings, diagnosed at 14.7, 10.1, and 3.2 years of age, demonstrated clinical improvement with weekly infusions of 2.0 mg/kg elosulfase alfa (Vimizim^®^, BioMarin Pharmaceutical, Novato, CA, USA). Patient 1 (oldest sibling) and Patient 2 (middle sibling) experienced a diagnostic delay of 8 years 7 months and 4 years after symptom onset, respectively. All three patients demonstrated improvements in growth, 6-min walk distance, joint range of motion, and respiratory function after 30 months of ERT. The treatment was well tolerated without any adverse events.

**Conclusions:**

This case series highlights the importance of early recognition of the clinical and imaging findings that are initially subtle in MPS IVA. Early treatment with ERT is necessary to slow irreversible disease progression and improve patient outcomes. The oldest sibling experienced improvements in mobility despite severe symptoms resulting from a late diagnosis. When evaluating patients with skeletal anomalies, imaging multiple body regions is recommended. When findings such as anterior beaking of vertebrae or bilateral femoral head dysplasia are present, MPS IVA should be included in the differential diagnosis. Newborn screening must be considered for early detection, accurate diagnosis, and initiation of treatment to reduce morbidity.

## Background

Mucopolysaccharidosis (MPS) IVA, also referred to as Morquio A syndrome, is a rare autosomal recessive lysosomal storage disorder caused by a deficiency in the enzyme N-acetylgalactosamine-6-sulfatase (GALNS) [1–4]. Impaired degradation of the glycosaminoglycans (GAGs) keratan sulfate and chondroitin 6-sulfate stemming from reduced GALNS activity results in undegraded GAGs in lysosomes across multiple tissues and organs, particularly in chondrocytes [3]. MPS IVA typically presents with bony abnormalities, short stature, and skeletal dysplasia [3, 5, 6]. Other findings include respiratory insufficiency, cardiovascular abnormalities, and dental impacts [5, 7]. Due to the progressive and heterogeneous nature of the disease and its variable presentation, early diagnosis can be challenging [1, 8]. However, prompt recognition, accurate diagnosis, and proper management of the disorder are necessary to improve patient outcomes [8].

Prior to the approval of elosulfase alfa (Vimizim^®^, BioMarin Pharmaceutical, Novato, CA, USA) as an enzyme replacement therapy (ERT) for patients with MPS IVA [9, 10], the treatment of MPS IVA mainly consisted of supportive therapy [11]. In a randomized, double-blind, placebo-controlled phase 3 pivotal trial that included patients ≥ 5 years of age, treatment with elosulfase alfa was well tolerated and resulted in a considerable improvement in endurance (6-min walk distance [6MWD]), substantial reduction in urinary keratan sulfate, and improvements in pulmonary function, height, and growth compared with placebo [11]. In a phase 2, open-label study in patients younger than 5 years of age, elosulfase alfa was well tolerated and resulted in a reduction of urinary keratan sulfate and a trend toward improvement in growth [12]. Previously published case reports of siblings with MPS disorders have shown that earlier treatment and management of these disorders results in improved outcomes [13–15].

Evidence of the impact of treatment with ERT and other interventions is needed to help inform healthcare providers of the importance of early diagnosis and to support newborn screening initiatives. In this case report, we describe three African American siblings who presented with similar symptoms at 5, 5, and 3 years of age, respectively. The two older siblings experienced a delay in diagnosis after the onset of symptoms, while the youngest sibling was suspected to have the same condition. The diagnosis for all three siblings was confirmed as MPS IVA via molecular genetic analysis and biochemical data. The differences in the siblings’ clinical courses highlight the importance of early recognition of MPS IVA because of the progressive nature of this disorder; specifically, understanding and recognizing the skeletal abnormalities of MPS IVA early will enable timelier diagnosis and treatment, which could help reduce mortality and morbidity.

## Methods

The three siblings with MPS IVA presented at Children’s Hospital of Philadelphia (CHOP) in the United States for clinical care. The siblings were born to non-consanguineous parents. Parental consent was obtained from both parents to use the patients’ medical data and photographs. All radiographs, magnetic resonance imaging (MRI), genetic testing, and other results were performed clinically and reviewed. All three patients initiated ERT (elosulfase alfa, BioMarin Pharmaceutical Inc., Novato, CA, USA) at the same time (Patient 1, 14.7 years of age; Patient 2, 10.1 years of age; Patient 3, 3.2 years of age).

## Results

### Clinical manifestations and laboratory evaluations

**Fig. 1:**
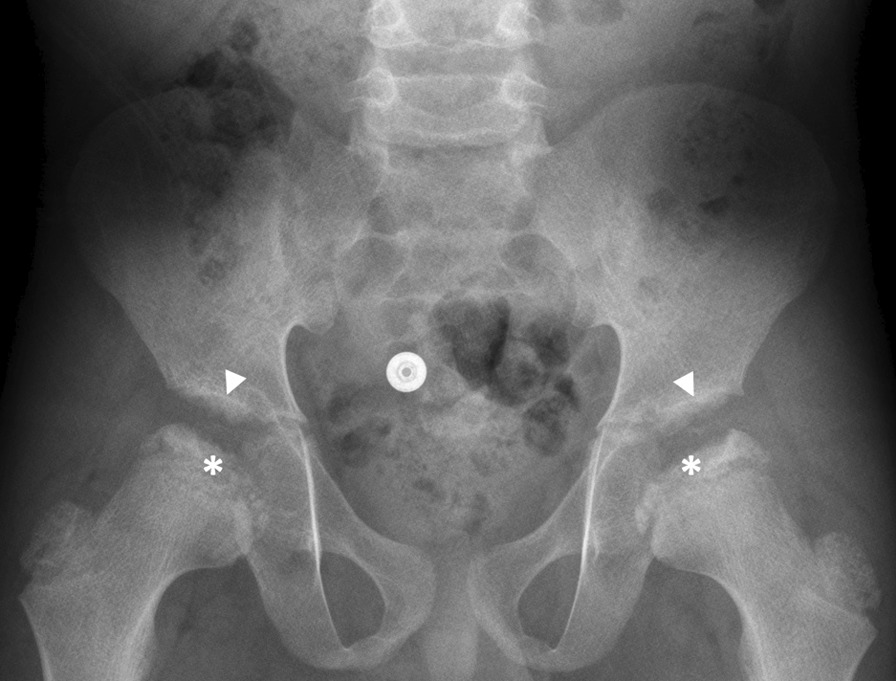
Anteroposterior radiograph of hips and pelvis in Patient 1 at 6 years of age shows bilateral sclerotic, fragmented, and flattened proximal femoral epiphyses (asterisks), irregular acetabula (arrowheads), and coxa valga deformities. The patient was initially diagnosed with Legg–Calve–Perthes disease; in retrospect, acetabular irregularities and coxa valga would not be typical in this condition

**Fig. 2:**
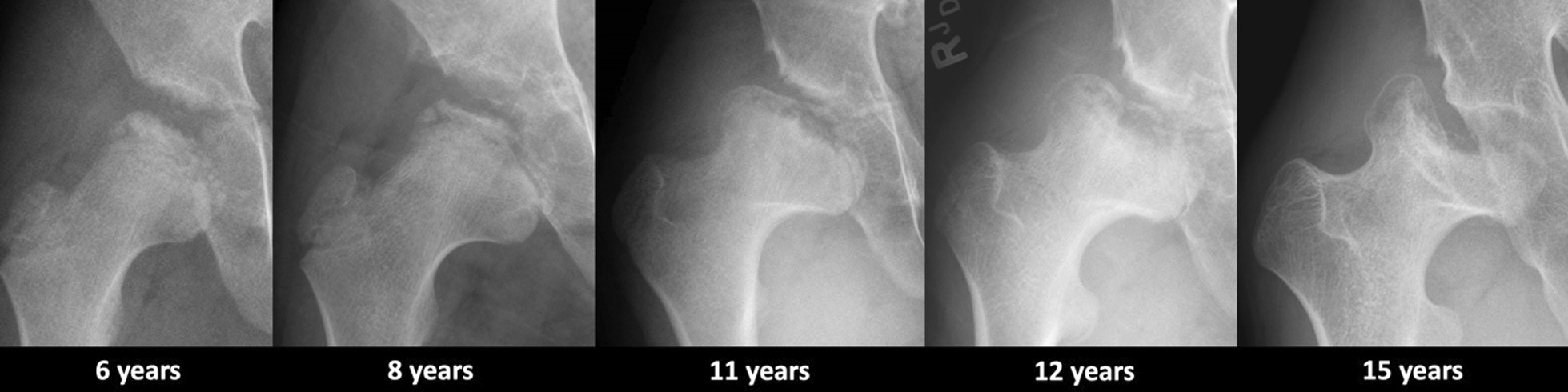
Representative serial radiographs of the proximal right femur of Patient 1 over time show progressive flattening of the epiphysis, concomitant widening of the proximal metaphyses, and increased irregularity of the acetabula resulting in compensatory pseudarthrosis-like formation

**Fig. 3:**
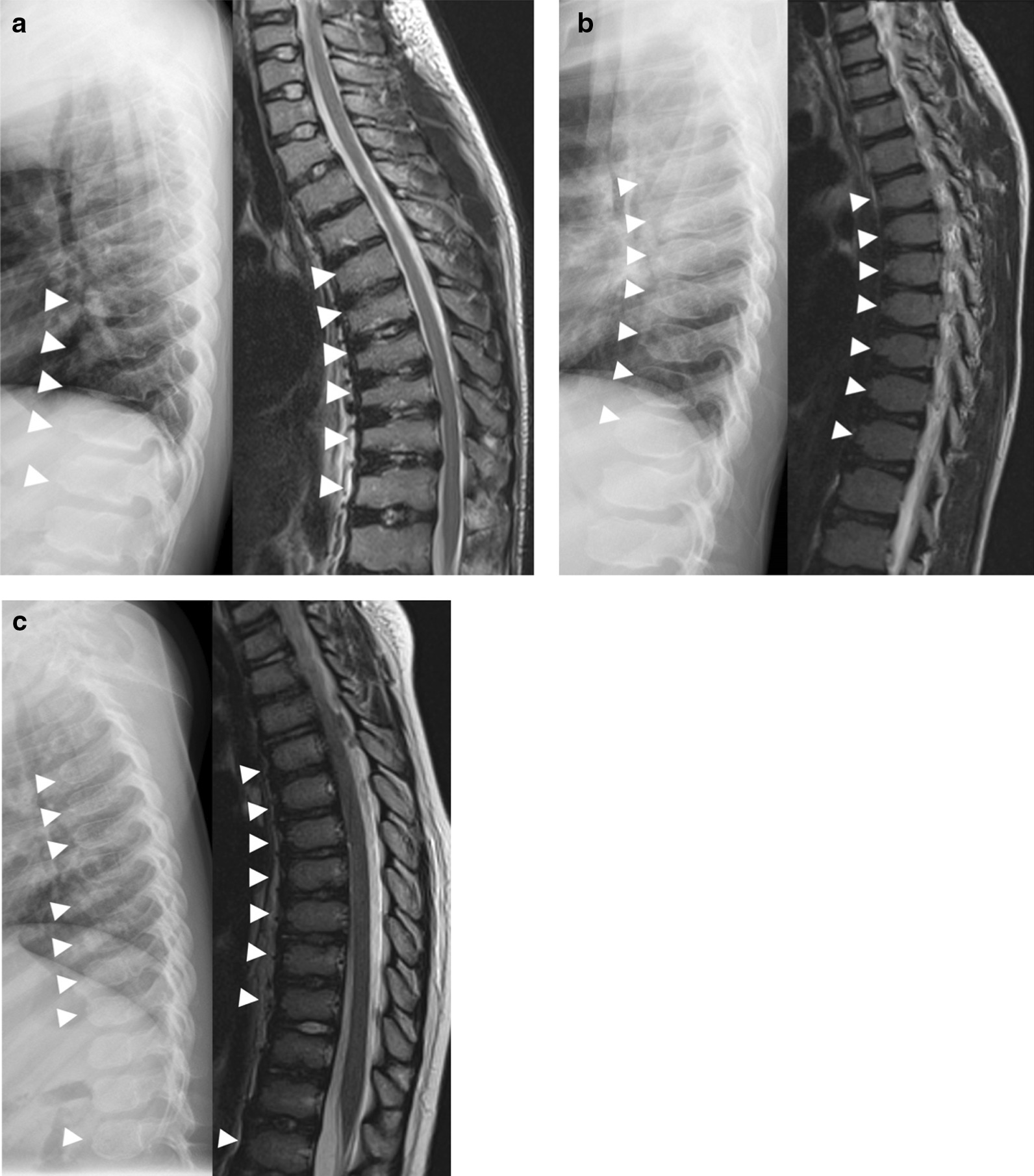
Lateral radiographs and MRIs of the thoracic spine in the three siblings. **a** In Patient 1, lateral thoracic spine radiograph (left) at 10 years of age shows relatively moderate vertebral body height loss (platyspondyly) and anterior vertebral body beaking, most prominent in the mid-to-lower thoracic spine (arrowheads). Sagittal T2-weighted MRI of the thoracic spine (right) shows moderate platyspondyly with prominent anterior beaking of multiple thoracic and upper lumbar vertebral bodies (arrowheads). The patient was initially diagnosed with spondyloepiphyseal dysplasia on the basis of clinical presentation, platyspondyly, and femoral epiphyseal abnormalities on imaging, although anterior beaking in the middle third of the spine is highly suggestive of MPS IVA, and vertebral flattening is not as prominent as would be expected in spondyloepiphyseal dysplasia. **b** In Patient 2, lateral thoracic spine radiograph at 6 years of age (left) shows anterior beaking of multiple thoracic vertebral bodies (arrowheads). Sagittal T2-weighted MRI of the thoracic spine at 10 years of age (right) shows anterior vertebral body beaking (arrowheads) with mild vertebral height loss without platyspondyly. Presence of anterior vertebral body beaking in this case is strongly suggestive of MPS IVA. **c** In Patient 3, lateral thoracic spine radiograph at 3 years of age (left) shows anterior beaking of multiple thoracic vertebral bodies without significant vertebral body height loss. Sagittal T2-weighted MRI of the thoracic spine at 5 years of age (right) highlights anterior vertebral body beaking. As with this patient’s siblings, anterior vertebral body beaking supported the diagnosis of MPS IVA

**Fig. 4:**
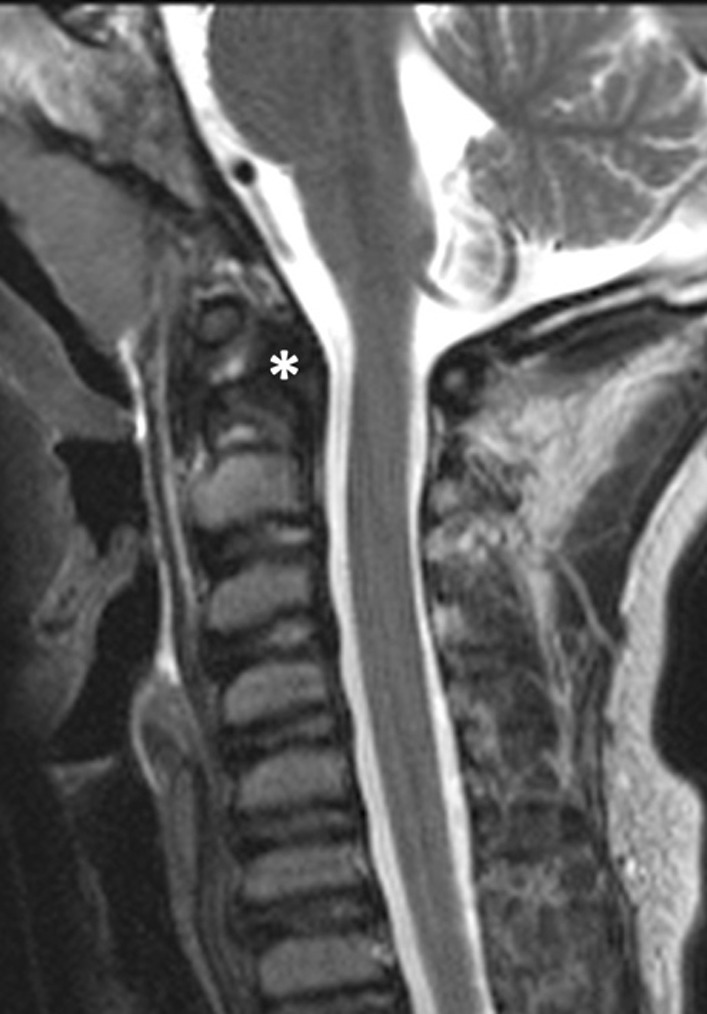
Sagittal T2-weighted TSE MRI of the cervical spine of Patient 1 at 10 years of age shows dysplastic odontoid process of C2, with irregular sclerosis and posterior migration of the tip of the dens (asterisk)

**Fig. 5:**
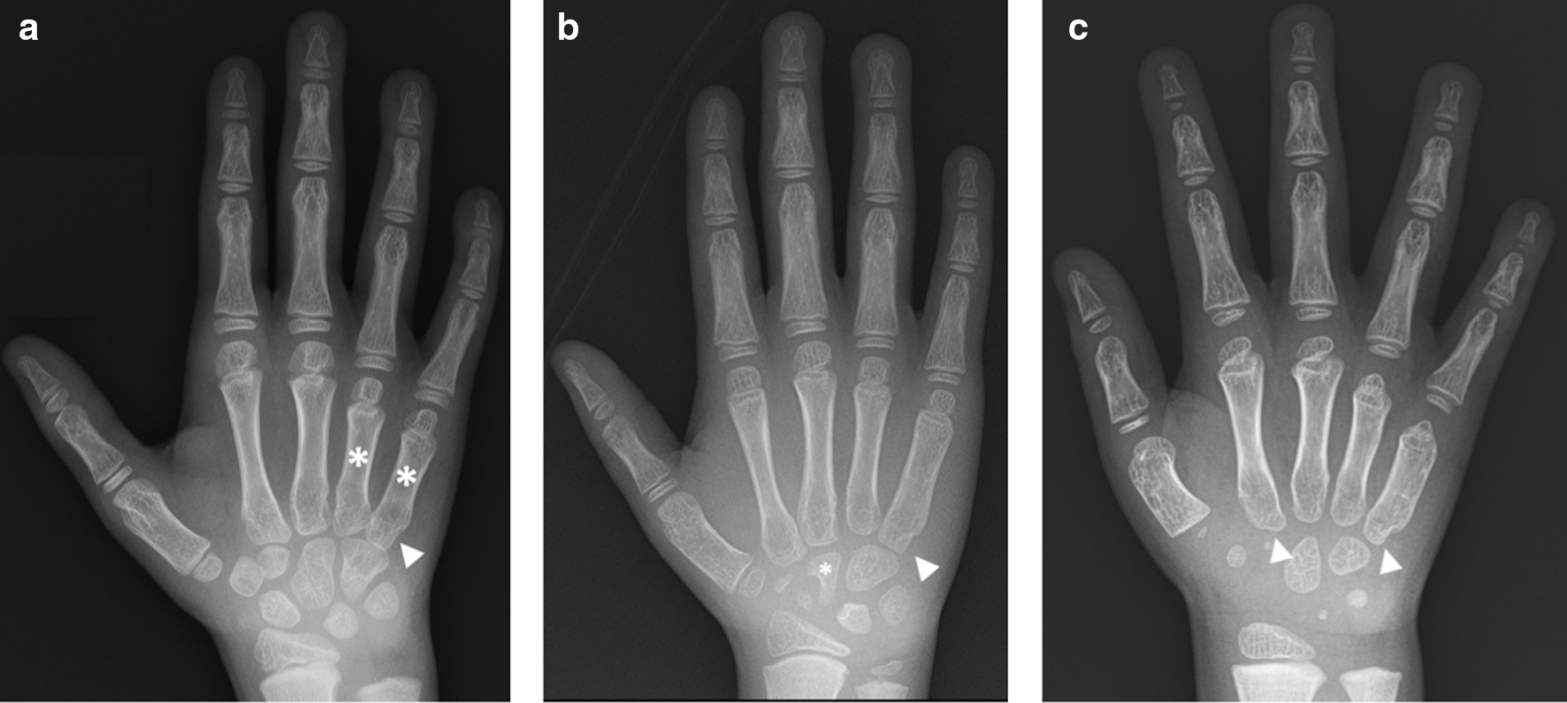
Radiographic features of the hand in the three siblings supportive of MPS IVA. **a** Patient 1 at 6 years of age showed prominent shortening of the fourth and fifth metacarpals (asterisks) with proximal pointing of the fifth metacarpal (arrowhead) and widening of the first metacarpal. **b** Patient 2 at 6 years of age showed proximal pointing of the fifth metacarpal (arrowhead) and irregular carpal bones (asterisk). Notably, this patient’s metacarpal abnormalities were much less conspicuous compared with those of his older brother (Patient 1) at the same age. **c** Patient 3 at 3 years of age showed proximally shortened, widened metacarpals with proximal pointing of the second and fifth metacarpals (arrowheads)

The clinical course of the three siblings is summarized in Table 1. All patients presented with similar symptoms at different ages. Patient 1 (oldest sibling) was noted to have a limp and endorsed hip pain at 5 years of age; he was diagnosed with bilateral Legg-Calve-Perthes disease (Fig. 1). At 7 years of age, he was referred to orthopedics for further evaluation and treatment at Children’s Hospital of Philadelphia (CHOP). Upon examination at the clinic, he presented with an abnormal wide gait with the lower legs placed in valgus; tenderness on the bilateral greater trochanter; and full range of motion, but with mild tenderness, on both knees and ankles. He underwent hip surgery and experienced a prolonged recovery with worsening of his symptoms. After his recovery, he was lost to follow-up and was seen again by orthopedics at CHOP at 11 years of age. At that time, he was unable to walk independently and was noted to have a wide-based stance with increased weakness. Pelvis radiographs over time demonstrated progressive destructive change of the hips (Fig. 2). His progressive weakness prompted a referral to the neuromuscular clinic, and electromyography findings were suggestive of mild myopathy. Radiographs and MRIs of the spine revealed multiple abnormalities, with prominent middle third spine vertebral involvement, consisting of prominent anterior beaking, posterior endplate concavities, and mild platyspondyly (Fig. 3). Spondyloepiphyseal dysplasia was suspected due to the combination of proximal femoral epiphyseal abnormalities, odontoid hypoplasia (Fig. 4), and platyspondyly, but genetic sequencing and deletion/duplication studies of *COL2A1* (collagen type II alpha 1) and *SEDL* (spondyloepiphyseal dysplasia tarda) were negative. In retrospect, other radiographic features, including anterior vertebral beaking and metacarpal abnormalities (Fig. 5), favored MPS IVA. At 5 years of age, Patient 2 (middle sibling) was noted to experience the same pattern of clinical signs and symptoms as Patient 1. A skeletal survey was completed, which showed symmetrically abnormal femoral heads, radial and ulnar epiphyses, and carpal bones. Additionally, the lumbar vertebrae were noted to be slightly flattened and bullet shaped. Following diagnosis of the two older brothers, Patient 3 (youngest sibling) was diagnosed through family screening. The parents observed his wide-based gait at around 2.5 years of age, but the symptom was not brought to medical attention until diagnosis of his older siblings.

**Table 1:** Clinical course for Patients 1, 2, and 3 with MPS IVA

	Patient 1	Patient 2	Patient 3
Current age	17 years, 8 months	13 years, 1 month	6 years, 2 months
Type of genetic analysis	Whole-exome sequencing	Whole-exome sequencing	Targeted *GALNS* sequencing
Results of *GALNS* gene analysis	c. 964G > C, p. A322P (paternally inherited, novel missense, variant of unknown significance)		
	c. 611A > G, p. N204S (maternally inherited, novel missense, variant of unknown significance)		
Age at onset of symptoms	5 years	5 years	3 years
Age at diagnosis	14 years, 7 months	10 years	3 years, 1 month
Age at initiation of ERT	14 years, 8 months	10 years, 1 month	3 years, 2 months

*ERT* enzyme replacement therapy, *MPS IVA* mucopolysaccharidosis type IVA

**Fig. 6:**
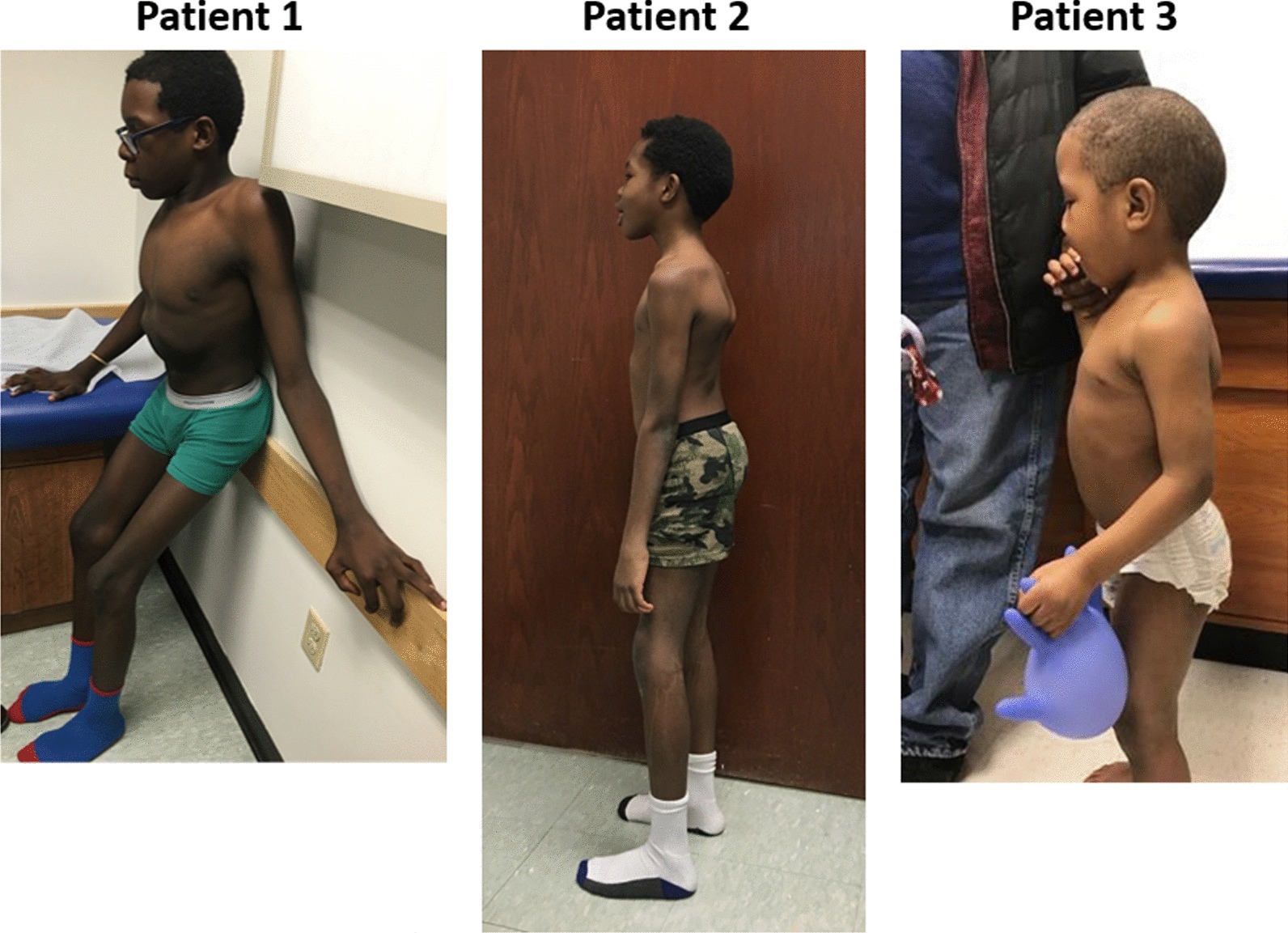
Physical examination of the siblings. Patient 1 at 14 years of age, Patient 2 at 9 years of age, and Patient 3 at 3 years of age. Lordotic posture can be noted in all three patients. Prior to starting ERT, all three siblings showed skeletal abnormalities and walking impairment, which were more advanced in the oldest sibling (Patient 1)

Patients 1 and 2 were referred for whole-exome sequencing at 14 and 9 years of age, respectively, which revealed compound heterozygous variants in the *GALNS* gene (Table 1). Enzyme testing revealed low GALNS activity. Urine GAG quantification revealed elevated GAGs, specifically chondroitin sulfate and keratan sulfate. Targeted-gene sequencing confirmed the diagnosis in Patient 3. Patient 3 was the least severely affected, with subtle differences in his skeletal survey and presenting with a slightly abnormal gait. The numerous skeletal abnormalities noted radiographically and reduced walking ability of Patient 1 highlight the progression of disease (Fig. 6).

The three siblings were started on intravenous infusions of elosulfase alfa at a dose of 2.0 mg/kg/week. The progression of clinical signs, symptoms, and laboratory studies in response to ERT after 30 months are summarized in Table 2.

**Table 2:** Comparison of clinical signs, symptoms, and laboratory studies of the three siblings at baseline and after 30 months of ERT

	Patient 1		Patient 2		Patient 3	
	Before ERT	After ERT	Before ERT	After ERT	Before ERT	After ERT
6MWD,^a^ m	50^d^	300	265	425	175^f^	290^e^
Height, cm (percentile^b^)	161.4 (25)	165.3 (7)	137.5 (44)	153.1 (43)	97.0 (60)	117.2 (74)
Weight, kg (percentile^b^)	50.8 (39)	65.3 (51)	27.9 (21)	37.1 (20)	16.6 (87)	27.3 (98)
BMI, kg/cm^2^ (percentile^b^)	19.5 (48)	23.9 (78)	14.8 (11)	15.8 (11)	17.6 (91)	19.9 (99)
Joint ROM, ^a^ degrees						
Hip abduction	R: 10	R: 20^e^	R: 20	–	–	R: 55
	L: 15	L: 20^e^	L: 20			L: 55
Hip internal rotation	R: 10	R: 10^e^	R: 30	R: 40^e^	–	R: 60
	L: 5	L: 15^e^	L: 30	L: 10^e^		L: 60
Knee extension	R: − 35	R: − 20	–	R: 0	–	R: 0
	L: − 55	L: − 30		L: − 15		L: 0
Ankle dorsiflexion with knee flexed	R: 8	R: 15^e^	–	–	–	–
	L: 10	L: 15^e^				
Ankle dorsiflexion with knee extended	–	R: 0	R: 8	R: 10	R: 10	R: 15
		L: 5	L: 5	L: 10	L: 10	L: 15
Popliteal angle	–	–	R: 15	R: 25	R: 30	R: 0
			L: 15	L: 35	L: 30	L: 0
Cardiac function^c^	No significant findings	–	No significant findings	No significant changes	No significant findings	–
Respiratory function						
FVC,^c^ %	150	157	71	85	–	–
FEV1,^c^ %	121	121	71	78	–	–
FEV1/FVC^c^	71	79	101	81	–	–
FEF_25–75_,^c^ %	76	70	41	46	–	–
Polysomnography	–	Snoring, no evidence of significant OSA	No evidence of OSA or restrictive lung disease	Normal breathing	No evidence of OSA or restrictive lung disease	Snoring, no evidence of substantial OSA
Ophthalmology	Mild corneal haze	Mild corneal haze	Mild corneal haze	Mild corneal haze	Subtle corneal haze	Diffuse fine corneal haze
Urinary GAGs						
Chondroitin sulfate, g/mol creatinine	7.77	2.87^e^	9.61	8.01^e^	21.2	14.19^e^
Keratan sulfate, μg/mol creatinine	5.35	2.97^e^	7.21	6.33^e^	16.6	9.95^e^

*6MWD* 6-min walk distance, *BMI* body mass index, *CDC* US Centers for Disease Control and Prevention, *ERT* enzyme replacement therapy,* FEF*_*25–75*_ forced expiratory flow between 25 and75% of FVC, *FEV1* forced expiratory volume in 1 s, *FVC* forced vital capacity, *GAG* glycosaminoglycan, *OSA* obstructive sleep apnea, *ROM* range of motion

^a^Initial joint ROM and 6MWD evaluations were conducted after initiation of treatment (Patients 1 and 2, 5 months; Patient 3, 3 months)

^b^CDC Clinical Growth Charts for 2–20 years of age

^c^Evaluation after 20 months of ERT

^d^Ambulation with bilateral forearm crutches in 59 s

^e^Evaluation after 30 months of ERT

^f^Distance walked in 2.75 min due to refusal to continue

### Endurance

After undergoing 30 months of ERT, all three patients showed an improvement in endurance, as measured by the 6-min walk test (6MWT). After 5 months of treatment, Patient 1 was unable to walk without the use of a walking aid and was only able to walk 50 m with the use of bilateral forearm crutches during the 6MWT. However, after 30 months of ERT, Patient 1 was able to walk unassisted for 300 m. The 6MWT results of Patients 2 and 3 were 265 m and 175 m, respectively, after 5 months of ERT. The 6MWT distances increased to 425 m after 30 months for Patient 2 and 290 m after 15 months for Patient 3.

### Clinical measures of skeletal growth and function

The height, weight, and BMI of all three patients increased from baseline after 30 months of ERT; of note, the largest increase in percentiles for both weight and height was found in Patient 3 (Table 2). Patient 1’s height was 165.3 cm (7th percentile), and his weight was 65.3 kg (51st percentile) 30 months after starting ERT. After starting ERT, he gained weight (from the 39th percentile to the 51st percentile), and his BMI increased (from 48th percentile to the 78th percentile). Although he gained 4 cm, his height percentile was not preserved, decreasing from the 25th to the 7th percentile. Patient 2’s height, weight, and BMI were preserved, with his height being 151.5 cm (43rd percentile), weight being 37.1 kg (20th percentile), and BMI being 15.8 kg/cm^2^ (11th percentile) at 30 months post-treatment. Patient 3’s height was 116.4 cm (74th percentile), his weight was 27.3 kg (98th percentile), and his BMI was 19.9 kg/cm^2^ (99th percentile) at 30 months post-treatment. The height percentile of Patient 3 increased from the 60th to the 74th percentile, his weight increased from the 87th to the 98th percentile, and his BMI increased from the 91st to the 99th percentile.

Joint range of motion (ROM) was assessed in the hips, knees, and ankles. Patient 1 experienced improvement (> 5°) in bilateral hip abduction, left hip internal rotation, bilateral knee extension, and bilateral ankle dorsiflexion with the knee flexed after 5–30 months of ERT; right hip internal rotation remained unchanged. In Patient 2, similar improvements were seen in left hip internal and external rotation, left ankle dorsiflexion with knee extended, and bilateral popliteal angles after 5–30 months of ERT. Improvements to right ankle dorsiflexion with knee extended were minor (< 5°), and right hip rotation was reduced (from 20° before ERT to 10° after 30 months of ERT for internal rotation and from 30° before ERT to 5° after 30 months of ERT for external rotation). In Patient 3, bilateral ankle dorsiflexion with knee extended ROM and bilateral popliteal angles improved after 30 months of ERT.

Prior to initiation of ERT, Patient 1 was unable to walk independently and unable to remain in standing position. Improvement in mobility was observed after 30 months of ERT, with the patient being able to walk without assistance for 300 m. Additional files 1 and 2 demonstrates this improvement in mobility.

### Cardiopulmonary evaluation

Initial cardiac evaluation was performed using an echocardiogram. There were no valve abnormalities or structural defects identified, and both left and right ventricles were of normal size with normal systolic shortening for all three patients before the initiation of ERT. There were no changes in their echocardiography findings or cardiac evaluations after 20 months of ERT.

Pulmonary function was evaluated using spirometry, and results were available for Patients 1 and 2. Prior to ERT, Patient 1 demonstrated 150% and 121% of predicted value for forced vital capacity (FVC) and forced expiratory volume in 1 s (FEV1), respectively. After 20 months of ERT, he had 157% and 121% of predicted value for FVC and FEV1, respectively. Patient 1’s FEV1/FEV was moderately increased and forced expiratory flow between 25 and 75% of FVC (FEF_25–75_) mildly decreased. Prior to ERT, Patient 2 had 71% of predicted value for both FVC and FEV1. After 20 months of ERT, his FVC and FEV1 predicted values increased to 85% and 78%, respectively. His FEV1/FEV was moderately decreased and FEF_25–75_ was mildly increased. Patient 2 reported experiencing shortness of breath during activity and excessive daytime somnolence when evaluated after 15 months of ERT. At 20 months of ERT, polysomnography showed snoring with no evidence of significant obstructive sleep apnea in Patients 1 and 3. Patient 2 had normal breathing during sleep.

### Ophthalmology

Both Patient 1 and Patient 2 had bilateral mild corneal haze prior to ERT, which remained unchanged after 30 months of ERT. In Patient 3, bilateral subtle corneal haze improved to diffuse, fine corneal haze after 30 months of ERT.

### Urinary glycosaminoglycan levels

In all three patients, urinary chondroitin sulfate (g/mol creatinine) and keratan sulfate (μg/mol creatinine) decreased with ERT (Table 2).

### ERT dosage and adverse reactions

All three patients received weekly infusions of elosulfase alfa at a dose of 2.0 mg/kg, and treatment was well tolerated throughout its duration without any adverse events.

### Clinical course of the three patients with MPS IVA

Patients with MPS IVA usually present with clinical signs or symptoms at the end of the first year of life. Most who present with classical symptoms are diagnosed at 2–3 years of age due to disproportional short stature, waddling gait, pectus carinatum, and frequent respiratory infections.

*Diagnostic delay:* Patient 1 presented with limp and hip pain around 5 years of age. He was seen by orthopedics at an outside institution and diagnosed with bilateral Legg-Calve-Perthes disease of unknown etiology. He was referred to orthopedics at the CHOP at 7 years of age, where a work-up for Legg-Calve-Perthes disease was undertaken and tested negative, including normal alpha globin, prothrombin, and Factor V Leiden genetic testing. His orthopedic surgeon questioned the diagnosis and referral to neuromuscular was recommended, given the progression of his symptoms and weakness. However, due to insurance issues, a consultation was not completed, and the patient was lost to follow-up for several years. He re-presented to care when his younger brother (Patient 2) developed the same symptoms. Further evaluation of the case by a neurologist and clinical geneticist lead to sending for whole-exome sequencing, which revealed compound heterozygous variants in the *GALNS* gene. In this case, the protracted diagnostic odyssey was likely due to the initial subtlety of findings and lack of knowledge about symptoms and signs of this rare, inherited metabolic disease among many physicians. Typically, disproportionately short stature has been considered a hallmark of this disorder. In this case series, Patients 1 and 2 were initially of normal height. Thus, a high index of suspicion and diligent examination of radiographs is required.

*Treatment response:* Patient 1 was severely affected at the time of diagnosis. He was unable to stand up without assistance and only capable of taking a few steps with assistance. After starting ERT, his endurance improved, and he was able to stand up independently; after 6 months of therapy, he started walking with crutches. One year after starting ERT, he started walking unassisted. Video 1 highlights his ability to walk unassisted during the 6MWT. His 6MWD increased significantly. His urine GAGs decreased, and his cardiac, pulmonary, and ophthalmological findings were stable. Due to the late diagnosis and severe skeletal involvement, his height increased only 4 inches in 30 months on ERT.

Patient 2 presented with a wide-based gait and hip pain at around 5 years of age. His symptoms remained more or less stable for the next 4 years, although each year, he had increased difficulty with walking. When he was diagnosed at 9 years of age, his 6MWD was 265 m, which increased to 425 m after 30 months of ERT. His walk is shown in Video 2. His height remained preserved around the 40th percentile.

Patient 3 was 3 years old at the time of diagnosis through family screening. Parents recalled that they noticed a wide-based gait beginning at around 2.5 years of age but were not concerned until his older brothers were diagnosed with MPS IVA. His waddling gait remained stable after the ERT. His 6MWD increased from 175 to 290 m, but the accuracy of this measurement was compromised because of poor cooperation. His height increased from the 60th to the 74th percentile.

## Discussion

The efficacy and safety of elosulfase alfa in treating MPS IVA have previously been demonstrated in patients ≥ 5 years and < 5 years of age. In this report, three siblings with MPS IVA initiated weekly infusions of elosulfase alfa starting at 14.7, 10.1, and 3.2 years of age, respectively. Thirty months of ERT was well tolerated and led to improvements in endurance (6MWD), growth, and joint ROM, and reduction in urinary GAGs, consistent with prior clinical studies.

Early diagnosis of MPS IVA is important to initiate treatment with ERT as soon as possible, as skeletal manifestations are considered irreversible. In this report, the three siblings received ERT at different stages of MPS IVA; Patient 1 had the most-severe presentation and was unable to walk independently at the start of treatment, whereas Patients 2 and 3 have developed some radiographic features of MPS IVA but have not yet experienced severe impairment to their mobility. While all three patients experienced improvement in clinical outcomes after 30 months of ERT, Patient 1 showed remarkable improvement in being able to walk unassisted, albeit not to the same level of his siblings who initiated treatment earlier. These three cases show us that ERT can reverse some progressive symptoms while slowing others.

The clinical presentation of MPS IVA is heterogeneous, and initial findings are subtle. This makes the diagnosis a challenge and can lead to substantial delays, as underscored by the diagnostic journey of Patient 1 [1, 8]. This case series highlights the importance of early recognition of the clinical and imaging findings of MPS IVA, especially considering the progressive nature of this disorder. The characteristic, progressive radiographic features in the pelvis, spine, and hand, as documented in this study, are key for MPS IVA diagnosis and follow-up testing. Thus, when evaluating patients with skeletal anomalies, imaging multiple body regions is recommended. With findings such as anterior beaking of vertebrae or bilateral femoral head dysplasia, MPS IVA should be included in the differential, as early treatment can reduce morbidity.

Despite the three siblings initially presenting with similar signs and symptoms, Patient 1 had a more-severe functional disability due to 9-year diagnostic delay. Improving awareness and recognition of MPS IVA will enable earlier diagnosis and treatment [8]. Evidence- and consensus-based management guidelines for MPS IVA for healthcare professionals have recommended implementation of newborn screening for MPS IVA to allow very early ERT to help slow the progression of the disease and improve outcomes [1]. Long-term ERT (elosulfase alfa, 2.0 mg/kg weekly IV infusions) is recommended in all patients with a confirmed MPS IVA diagnosis [1].

## Conclusion

Treatment with elosulfase alfa ERT in three siblings with MPS IVA was effective in improving clinical outcomes and was well tolerated. The variable clinical presentation, delay in diagnosis, and progressive nature of MPS IVA, as demonstrated by these three siblings, highlight the importance of improving awareness and recognition of the disease’s early signs and symptoms. The initiation of ERT in these siblings improved their endurance, reduced their pain, and improved their ranges of motion. In the younger siblings, height was preserved or improved. Additionally, in the youngest sibling, ophthalmologic findings improved. The youngest and middle siblings continue to walk independently, and the oldest sibling can now walk with crutches or unassisted for 300 m. This highlights the substantial effect of ERT in slowing disease progression and improving patient outcomes even in the late-diagnosed patient. Newborn screening for MPS IVA is necessary to prevent diagnostic delay and initiate treatment as early as possible to achieve better clinical outcomes.

## Supplementary information

**Additional file 1:** The patient 1’s 6 minute-walk test at 17 year 8 months of age on ERT.

**Additional file 2:** The patient 2’s 6 minute-walk test at age 13 year 1 month on ERT.

## Data Availability

All data generated or analyzed during this study are included in this published article (and its Additional files 1 and 2).

## Abbreviations

6MWD: 6-Min walk distance
6MWT: 6-Min walk test
BMI: Body mass index
CHOP: Children’s Hospital of Philadelphia
COL2A1: Collagen type II alpha 1
ERT: Enzyme replacement therapy
FEF_25–75_: Forced expiratory flow between 25 and 75% of FVC
FEV1: Forced expiratory volume in 1 s
FVC: Forced vital capacity
GAGs: Glycosaminoglycans
GALNS: *N*-Acetylgalactosamine-6-sulfatase
MPS: Mucopolysaccharidosis
MRI: Magnetic resonance imaging
ROM: Range of motion
SEDL: Spondyloepiphyseal dysplasia tarda
